# Study on the Accumulation, Excretion, and Distribution of Cadmium in Mice After Dietary Exposure to Cadmium-Rich Swimming Crab *Portunus trituberculatus*

**DOI:** 10.3390/foods15162839

**Published:** 2026-08-14

**Authors:** Rui He, Xiao Mo, Qi Li, Minming Yan, Yongfu Shi, Nana Sun, Ruolin Li, Xuanyun Huang, Liangliang Tian, Feng Han, Siman Li

**Affiliations:** 1 Key Laboratory of Oceanic and Polar Fisheries Ministry of Agriculture and Rural Affairs P. R. China, East China Sea Fisheries Research Institute, Chinese Academy of Fishery Sciences, Shanghai 200090, China; 2 College of Food Science and Technology, Dalian Ocean University, Dalian 116023, China; 3 College of Food Science and Technology, Shanghai Ocean University, Shanghai 201306, China; 4 Shanghai Pudong District Agricultral Product Quality and Safety Center, Shanghai 201201, China

**Keywords:** cadmium, *Portunus trituberculatus*, dietary exposure, tissue accumulation

## Abstract

To investigate the patterns of cadmium accumulation and distribution in mice following oral ingestion of cadmium-enriched *Portunus trituberculatus*, this study selected 4-week-old ICR mice as a model organism. A control group was fed a standard diet, while an exposure group was fed a diet supplemented with 10% cadmium-enriched *Portunus trituberculatus*. After 28 consecutive days of exposure, the mice were switched to a standard diet for 7 days. Tissues and feces were collected throughout the experiment; cadmium levels were determined by inductively coupled plasma mass spectrometry (ICP-MS) following microwave digestion. The results showed that after 28 days of exposure, cadmium accumulation was observed in the kidneys (females: 0.046 ± 0.001 mg/kg, males: 0.053 ± 0.005 mg/kg) and livers (females: 0.046 ± 0.008 mg/kg, males: 0.034 ± 0.001 mg/kg), while levels in the control group were below the limit of detection; After a subsequent 7-day period of feeding standard diet, cadmium accumulation was observed in the kidneys (females: 0.070 ± 0.001 mg/kg, males: 0.047 ± 0.006 mg/kg), livers (females: 0.040 ± 0.001 mg/kg, males: 0.035 ± 0.009 mg/kg), and the intestines (females: 0.028 ± 0.006 mg/kg, males: 0.029 ± 0.001 mg/kg) still contained trace amounts of cadmium that had not been excreted. An apparent mass balance analysis suggested that more than 90% of ingested Cd was not retained in the analyzed tissues. In summary, although cadmium ingested orally is primarily excreted, the clearance rate for cadmium accumulated in the liver and kidneys of mice is relatively low, and residual levels remain in the tissues even 7 days after exposure ceased, indicating that even when the majority of ingested Cd is eliminated, the fraction retained in target organs may persist after exposure cessation and warrants further investigation under longer-term exposure scenarios.

## 1. Introduction

Cadmium (Cd) is an environmental pollutant with strong carcinogenic and mutagenic properties. Exposure to Cd primarily occurs through two routes: dietary and environmental. Among these, dietary intake is the main route of Cd exposure for the general population, accounting for 90% of total Cd exposure [1]. Once Cd enters the human body, its absorption rate in the gastrointestinal tract is typically less than 5–10%. However, due to its long biological half-life of 20–30 years, even long-term intake of trace amounts can lead to progressive accumulation in organs such as the liver and kidneys [2,3]. This, in turn, triggers chronic health effects such as liver and kidney dysfunction, bone metabolism abnormalities, and cardiovascular damage [4,5,6,7]. The International Agency for Research on Cancer (IARC) has explicitly classified Cd and its compounds as Group I human carcinogens [8]. Therefore, understanding the absorption, distribution, and retention characteristics of Cd from different dietary sources is essential for accurately evaluating dietary Cd exposure.

*Portunus trituberculatus*, as a frequently consumed aquatic product by coastal residents during certain seasons, has a strong ability to accumulate Cd and is one of the potential sources of dietary exposure. However, current research on the health risks of cadmium has largely focused on plant-based foods such as rice and wheat that are contaminated through soil pollution; relatively few studies have examined the bioavailability and toxicokinetic behavior of cadmium derived from aquatic food sources. Dietary exposure to Cd is commonly studied using mouse/rat models to investigate the toxic effects of oral exposure to food in order to assess the health impact of such foods on humans. Yang et al. [9] fed both non-Cd-contaminated and Cd-polluted rice (0.005 mg/kg and 0.74 mg/kg, respectively) to female and male mice for one month. The results revealed that Cd exhibits specific toxicity to male mice, with higher Cd accumulation observed in the liver and femur of male mice compared to females, and a greater risk of osteoporosis was also observed in male mice. Zhu et al. [10] investigated the bioavailability of arsenic (As), Cd (Cd), and lead (Pb) in mice following oral administration of Okinawan abalone for 20 days, and determined the total uptake rate of toxic elements in liver and kidney tissue samples as well as blood. The results of the in vivo experiment indicate that the oral bioavailability of As, Cd, and Pb is approximately 0.33%, 0.45%, and 0.74%, respectively. Currently, there is limited research on Cd exposure using aquatic products as a food matrix in mouse/rat models. According to the National Food Safety Standard: Maximum Limits for Contaminants in Food (GB 2762-2017) [11], the maximum Cd content in crustacean aquatic products is 0.5 mg·kg^−1^; however, the new national standards for 2022 and 2025 have specifically adjusted this limit to 3.0 mg·kg^−1^ for sea crabs. Although the limit has been relaxed, the 2025 China Fisheries Statistical Yearbook [12] indicates that China’s production of Chinese blue swimming crabs reached 456,000 metric tons in 2024, accounting for 70% of all wild-caught crabs. Given the high consumption rates among coastal residents during specific seasons and the strong Cd bioaccumulation characteristics of Chinese blue swimmer crabs, the risk of dietary Cd exposure from crustaceans cannot be overlooked. Therefore, conducting in-depth research on Cd exposure caused by aquatic products in diets is of critical importance for the health of coastal residents.

In previous studies on Cd exposure, high concentrations of inorganic Cd were typically added to the exposure matrix to obtain more pronounced experimental results. This differs from the forms of Cd typically accumulated in general food matrices and does not accurately reflect the actual accumulation and excretion of Cd in mice and rats following dietary intake, potentially leading to an overestimation of the health risks associated with dietary Cd exposure from such matrices. Therefore, this study used mice as an experimental model to systematically elucidate the tissue distribution characteristics, accumulation tendencies in target organs, and elimination kinetics following oral ingestion of cadmium-enriched swimming crabs. This study aims to address the lack of baseline data on dietary cadmium exposure from seafood, provide experimental evidence regarding the toxicokinetic behavior of cadmium from seafood sources, and establish preliminary information on dietary cadmium exposure associated with the consumption of crustacean seafood.

## 2. Materials and Methods

### 2.1. Instruments and Reagents

Nitric acid (analytical purity, J. T. Baker Company, Radnor, PA, USA); Pentobarbital sodium anesthetic (Shanghai Yuanye Biotechnology Co., Ltd., Shanghai, China); Cd standard solution (10 µg/mL), Indium (In) internal standard solution (10 µg/mL) (Agilent Technologies, Santa Clara, CA, USA).

Agilent-7500 Inductively Coupled Plasma Mass Spectrometer, Agilent Technologies, Santa Clara, CA, USA; HP-H35SC Electric Heating Plate, Shengda Jieshen Automation Equipment Co., Ltd., Jinan, China; Ethos 1/A Microwave Digestion System, Milestone, Sorisole, Italy; Milli-Q ultrapurewater purification system, Millipore, Burlington, MA, USA.

### 2.2. Experimental Animals

This study used 4-week-old ICR mice as experimental animal models, totaling 138 mice (69 females and 69 males, from the Shanghai Laboratory Animal Research Center, weighing 20 ± 2 g). After one week of acclimatization, the animals were randomly divided into a Cd-exposed group (66 mice) and a control group (72 mice) according to the experimental design, with males and females housed in separate cages. Mice in the exposure group were fed a diet containing a mixture of edible tissues from Cd-enriched swimming crabs as the dietary source of exposure for 28 consecutive days to evaluate the absorption, distribution, and accumulation characteristics of Cd from seafood in mammals. At the end of the exposure period, the diet of the final batch of mice in the exposure group was switched to standard chow, and they were continued to be housed for 7 days to observe Cd residual levels and clearance following cessation of exposure.

During the experiment, the mice were housed in an SPF-grade animal facility, with the ambient temperature maintained at 22 ± 2 °C and relative humidity at 50 ± 10%. The experimental animals were managed according to standardized protocols, receiving feed at fixed times and in fixed quantities each day, and the housing environment was cleaned and bedding replaced prior to each feeding. All animals were randomly assigned to groups, and the experiment was conducted in strict accordance with ethical guidelines for laboratory animals.

### 2.3. Feed Preparation

The *Portunus trituberculatus* purchased from the Zhoushan wharf was dissected, and the edible tissues were mixed together. They were then dried in an air-blowing oven at 100 °C, ground into a powder using a mill, and the same process was applied to the ordinary feed. Subsequently, the two powders were mixed in proportion to produce granulated feed. Before the formal exposure experiment, a quantitative analysis of total Cd was conducted on the exposed feed and the ordinary feed to determine the total Cd intake of the experimental mice.

### 2.4. Experimental Plan

During the experiment, mice in the exposure group were fed a diet containing 10% swimming crab feed enriched with Cd to establish a dietary exposure model for Cd from seafood sources. The control group was fed a standard diet without added crab. Prior to the start of the exposure experiment, the mice underwent a one-week acclimation feeding period, during which daily feed intake was monitored to determine the appropriate feeding amount for subsequent exposure.

During the 28-day exposure period, feed was provided daily based on the average consumption during the acclimation period to ensure comparable dietary intake among animals within the same sex group. The Cd exposure dose was calculated based on the Cd concentration measured in the experimental diet and the individual body weight. At the end of the exposure period, mice in the exposed group were switched to a standard diet for an additional 7 days as a washout period. Tissue and fecal samples were then collected to evaluate the accumulation and excretion patterns of Cd derived from swimming crabs in the mice.

### 2.5. Collection and Preparation of Organizational Sample

Before the start of the exposure experiment, three male and three female mice were selected to measure their pre-exposure Cd levels. During the experiment, samples were collected 12 h, 24 h, 48 h, 72 h, 5 d, 7 d, 10 d, 14 d, 21 d, and 28 d after feeding the Cd-containing diet to determine the accumulation of total Cd in various organs and tissues, as well as its excretion, following the mice’s consumption of spiny crabs. After 28 days of exposure, the mice in the exposed group were fed a standard diet for one week, and all remaining mice were dissected and sampled on day 35 after the start of the exposure experiment.

Two hours prior to sampling, 12 mice were randomly selected (exposed group: 3 males and 3 females; control group: 3 male and 3 female) and placed in specially designed cages with open bottoms for 2 h to collect feces. Before dissection began, the mice were anesthetized, blood was drawn from the eyeball, and after blood collection, the mice were euthanized by snapping their necks. During dissection, 10 tissues were collected, including the brain, heart, lungs, liver, stomach, spleen, intestines, kidneys, gonads, and muscle. The tissues were then homogenized and stored in a freezer at −20 °C.

### 2.6. Sample Pretreatment and Analysis

Total Cd in the samples described in Section 2.5 was determined and analyzed using Method 1 of GB 5009.268-2025 [13]: Inductively Coupled Plasma Mass Spectrometry (ICP-MS). In accordance with the standard, the samples were weighed and digested; the digestion procedure is shown in Table 1. For the quantification of Cd, seven concentrations of standard working solutions (0, 1, 2, 5, 10, 20, and 50 µg/L) were used as internal standards. The R^2^ value of the calibration curve was 0.9998. The limit of detection (LOD) for this method was 0.02 mg/kg, and the limit of quantification (LOQ) was 0.05 mg/kg.

### 2.7. Quality Control

To ensure analytical quality, *Sinonovacula constricta* reference materials (certified by the Second Institute of Oceanography, State Oceanic Administration) were analyzed alongside the samples. The QC procedure, which included assessments of precision, repeatability, and trueness, was repeated three times to guarantee accurate and reliable quantification of total Cd. Data are expressed as means ± SD.

### 2.8. Data Analysis

All statistical analyses were performed using SPSS 27.0 software. All data are reported as mean ± standard error (SE). All significance tests were conducted using an independent samples *t*-test assuming a normal distribution. A *p*-value < 0.05 was considered to indicate a significant difference in means. Graphs were primarily created using Origin 2024.

## 3. Results and Discussion

### 3.1. Quality Control Results

The total Cd content in the *Sinonovacula constricta* reference material was determined by ICP-MS to be 0.37 ± 0.01 mg/kg, which is in good agreement with the certified value of 0.38 ± 0.02 mg/kg. This confirms the accuracy and reliability of the proposed method.

### 3.2. Daily Dietary Exposure in Mice

The Cd content in the edible tissue of the swimming crabs was 290.8 ± 2.8 mg/kg (dry weight). The Cd content in the exposed diet was 30.8 ± 3.06 mg/kg (dry weight), while that in the standard diet was 1.51 ± 0.018 mg/kg (dry weight). One week before the exposure experiment, the mice were fed a diet at a rate of 250.0 g/kg body weight (bw)/day. Observations of feeding behavior revealed that female mice left 143.5 g/kg bw/day of feed, while male mice consumed the entire 250.0 g/kg bw/day. To ensure consistent daily exposure doses among mice of the same sex, the feed ration for female mice was adjusted to 150.0 g/kg bw/day at the onset of the formal exposure experiment, while that for male mice remained unchanged. The proportion of the Cd-spiked diet in the mixture was maintained at 10%, homogeneously mixed with the standard chow. Consequently, the daily Cd intakes via normal feeding were 650.0 ± 34.4 µg/kg bw/day for exposed females, 1140.0 ± 72.5 µg/kg bw/day for exposed males, 211.0 ± 27.6 µg/kg bw/day for control females, and 352.0 ± 46.0 µg/kg bw/day for control males.

### 3.3. Accumulation of Total Cd in Mice

**Figure 1:**
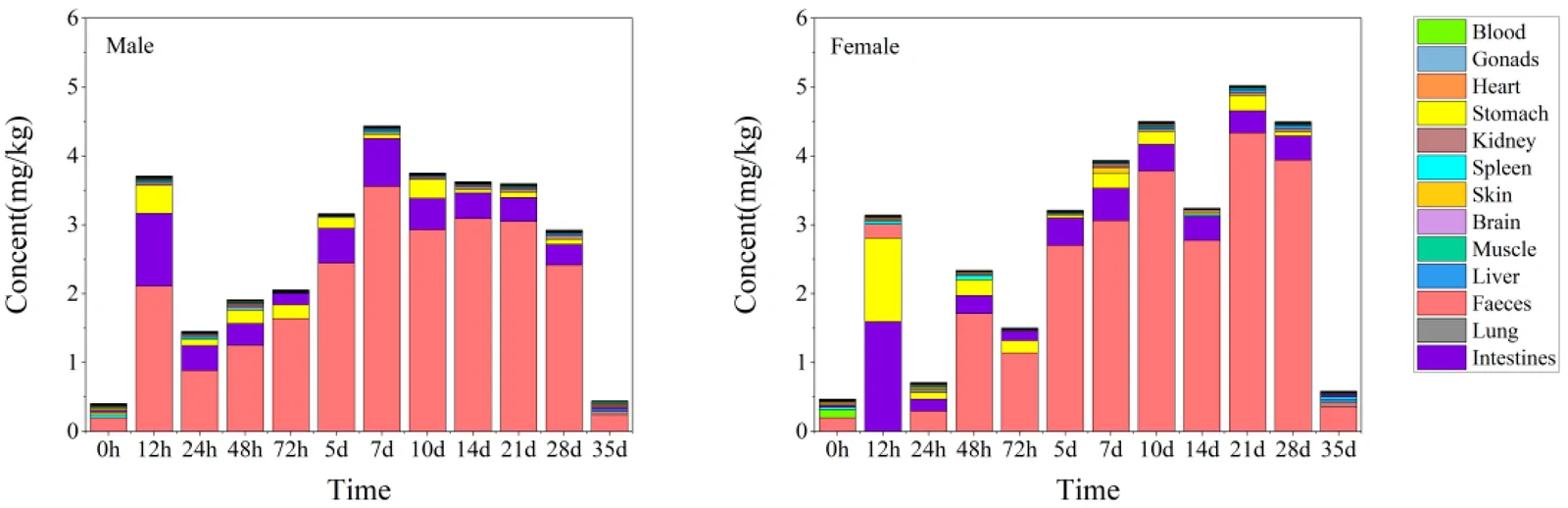
Concentrations of Cd in various tissues and feces of mice in the exposed group under different exposure times.

Figure 1 shows the Cd concentrations in various tissues and feces of mice in the exposed group at different time points following consumption of feed containing Cd-enriched swimming crabs. At 12 h post-exposure, the highest Cd concentration was found in the intestines of male mice (2.46 ± 0.003 mg/kg), followed by feces (1.06 ± 0.002 mg/kg) and the stomach (0.406 ± 0.001 mg/kg). Cd was detected in the kidneys at trace levels below the LOQ, while concentrations in all other tissues were below the LOD. In female mice at 12 h post-exposure, Cd concentrations were highest in the intestines (1.60 ± 0.015 mg/kg). Concentrations in the stomach (1.21 ± 0.001 mg/kg) were higher than those in feces (0.207 ± 0.005 mg/kg). Trace amounts of Cd were detected in the kidneys and spleen, with concentrations in all other tissues remaining below the LOD.

After 24 h of exposure, the total Cd content in various tissues and organs of male mice was as follows: feces > intestines > stomach > spleen ≈ brain > kidneys ≈ heart ≈ lungs ≈ liver ≈ gonads ≈ muscle. Compared with the 12 h exposure period, Cd concentrations in the feces, intestines (0.362 ± 0.201 mg/kg), and stomach (0.09 ± 0.001 mg/kg) of male mice all showed a downward trend, which is presumed to be related to the excretion of some Cd via the digestive tract and its elimination in the feces. In female mice, Cd concentrations in feces increased after 24 h of exposure (0.298 ± 0.011 mg/kg) compared to 12 h, while Cd concentrations in the intestines and stomach also showed a decreasing trend. In addition, trace amounts of Cd were detected in the lungs and fur of female mice, while concentrations in other tissues and organs were below the limit of detection.

At 48 h of exposure, similar to the trend observed at 24 h, Cd concentrations were highest in the feces of both male and female rats, followed by the intestines and stomach. At this point, Cd levels in the spleen and brain of male rats had dropped to undetectable levels, while trace amounts of Cd (0.048 ± 0.006 mg/kg) were detected in the gonads; in female rats, Cd levels in the lungs and fur had also dropped to undetectable levels, while Cd (0.067 ± 0.0823 mg/kg) was detected in the spleen.

After 72 h of exposure, Cd was still highest in the feces of both male and female mice. Unlike at 48 h, the Cd content in the stomach was slightly higher than in the intestines, while Cd was not detected in the other tissues. After 5 days of exposure, the Cd content in the feces of both male and female mice remained the highest, followed by the intestines and stomach, with no Cd detected in the remaining tissues.

**Figure 2:**
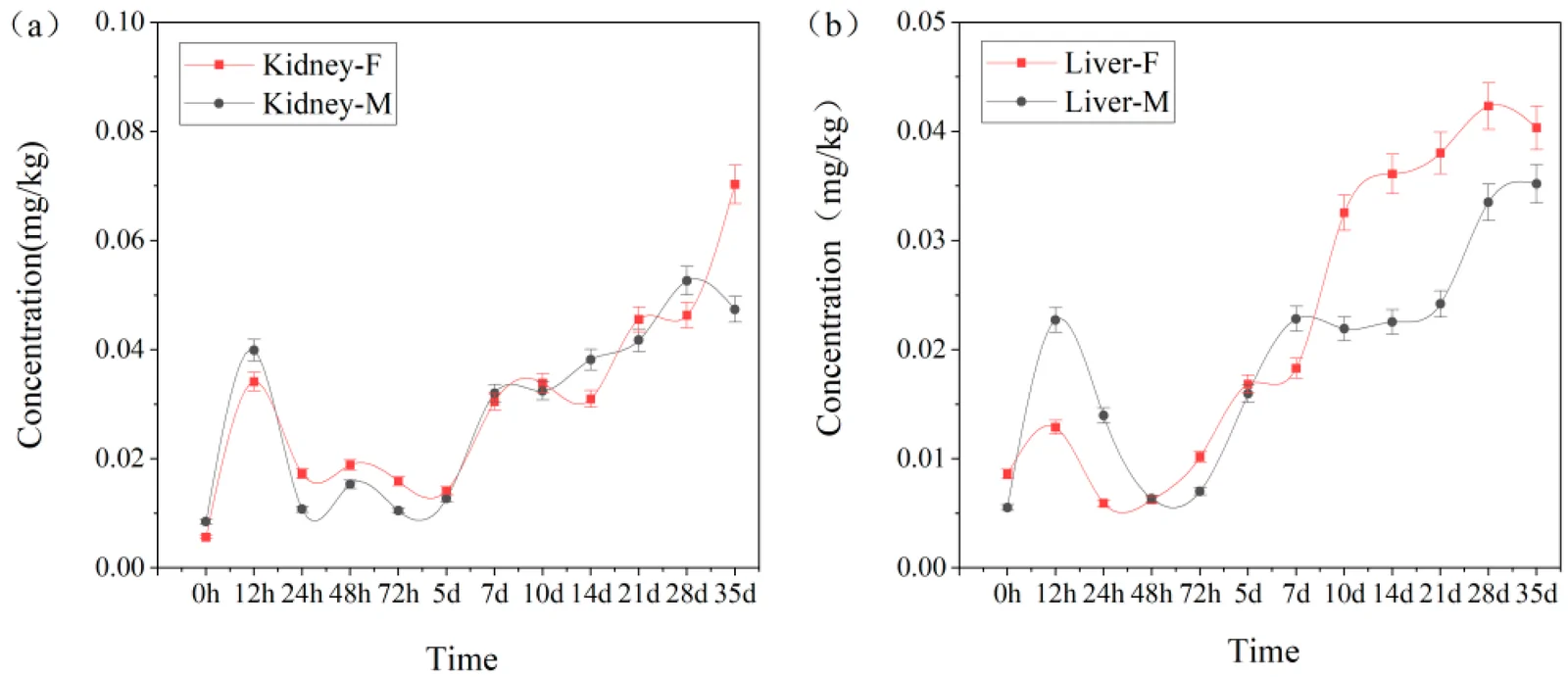
Cd accumulation in mouse kidney (**a**) and liver (**b**) at different exposure times.

From day 7 to day 28 of exposure, Cd levels were consistently highest in the feces of all mice, followed by the intestines and stomach. During this period, trace amounts of Cd were continuously detected in the kidneys and liver, but most did not reach the limit of quantification. By day 28, Cd levels in the kidneys of male mice reached the limit of quantification (0.0527 ± 0.005 mg/kg), indicating a slow accumulation in the kidneys of male mice during nearly one month of continuous exposure; although Cd levels in the kidneys of female mice still did not reach the limit of quantification, their Cd response values gradually increased over time, also showing a trend of slow accumulation. The pattern of Cd accumulation in the liver was similar to that in the kidneys, with response values increasing over time but remaining below the limit of quantification (Figure 2).

**Figure 3:**
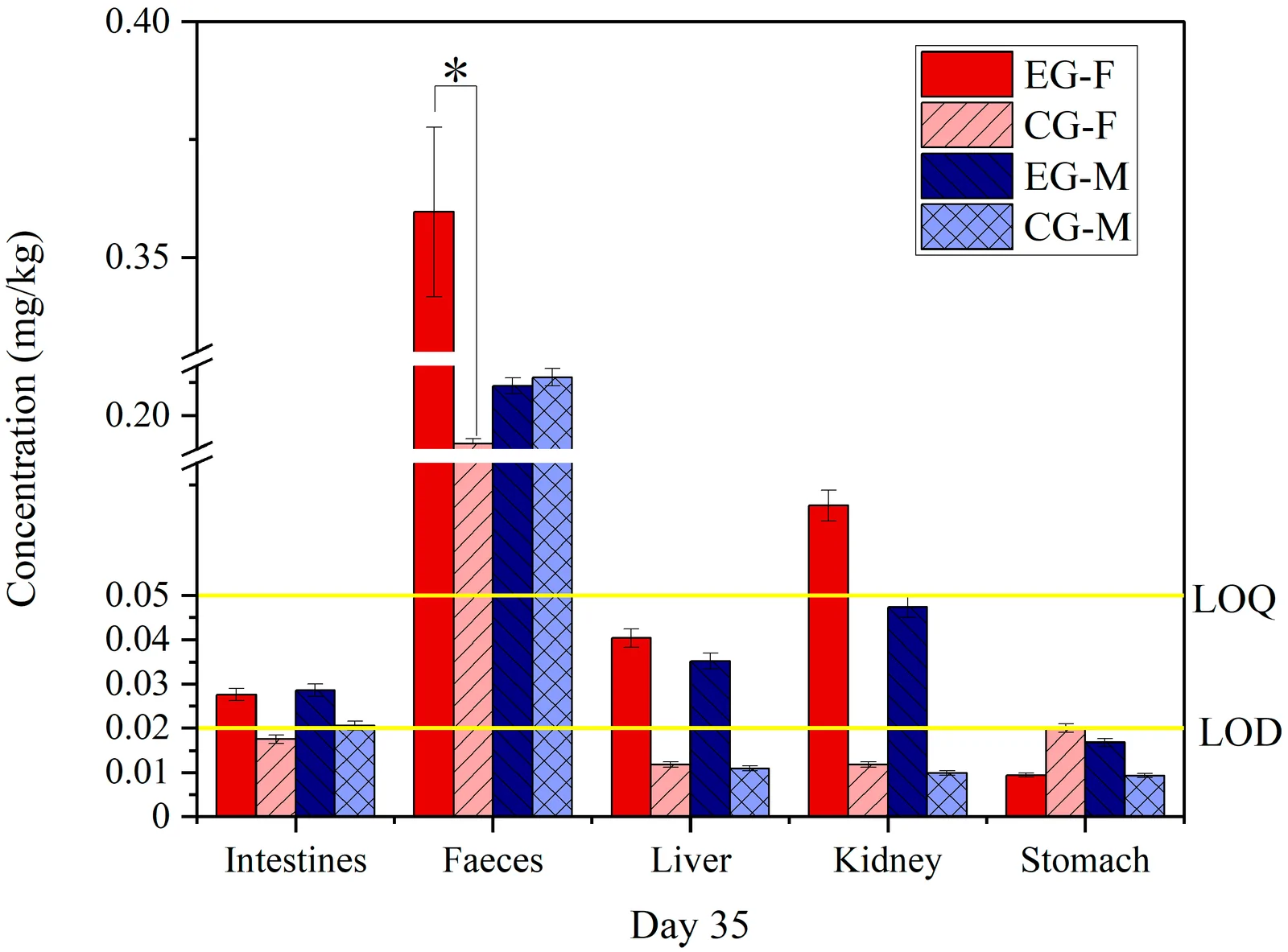
Accumulation of Cd in the intestine, faeces, liver, spleen, kidney and stomach of mice on day 35 (EG: Exposure group; CG: Control group; F: Female; M: Male). “*” indicates statistical significance.

On day 35, Cd was detected in the intestines, feces, liver, kidneys, and stomach of the mice, as shown in Figure 3. Cd concentrations were highest in feces among all tissues. In female mice, Cd concentrations in the feces of the exposed group were significantly higher than those in the corresponding control group (*p* < 0.05); in male mice, there was no significant difference in fecal Cd levels between the exposed group and the corresponding control group. Cd levels in the kidneys were second only to those in feces, representing the highest concentration among all tissues at this time point. Furthermore, Cd levels in the kidneys of both male and female mice in the exposed group were significantly higher than those in their respective control groups, indicating that even after one week of returning to a normal diet, the trace amounts of Cd accumulated in the kidneys had not been completely excreted.

### 3.4. The Cd Burden in Mice Feces

**Figure 4:**
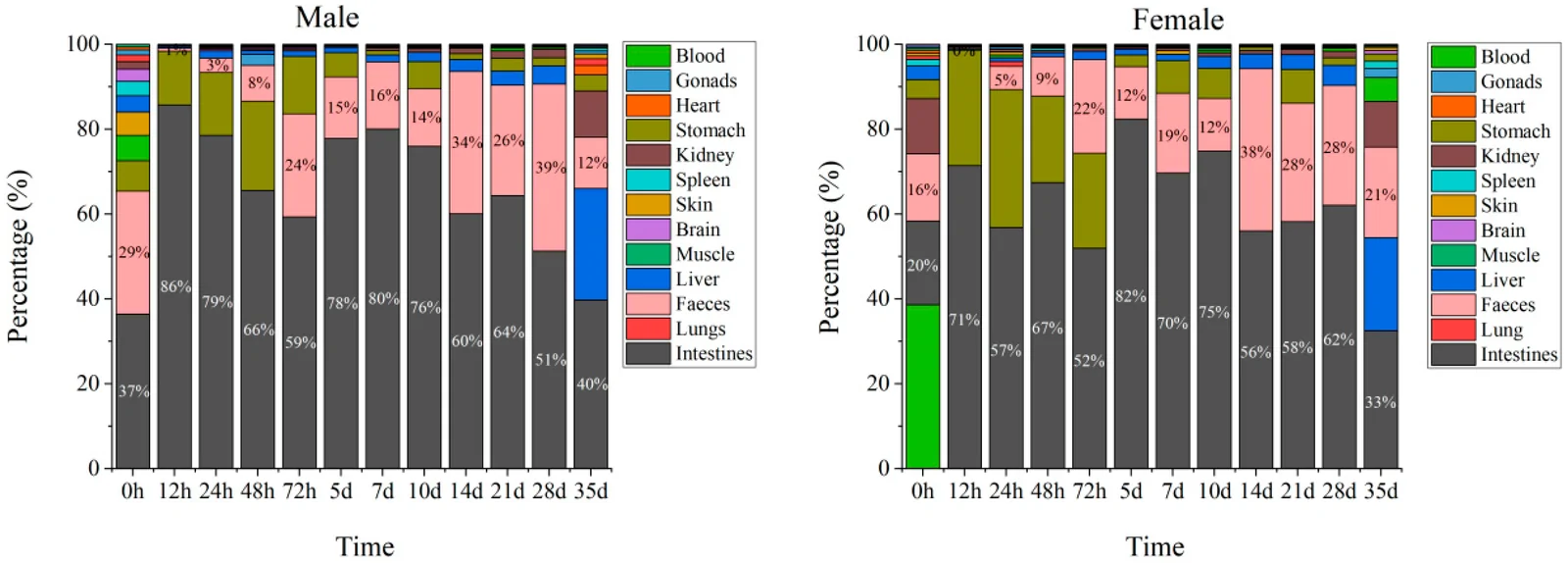
Different exposure time groups showed varying accumulation percentages of Cd in mouse tissues and feces.

Figure 4 shows the stacked percentage graphs of Cd burdens in various tissues and feces of female and male mice at different time points after dietary Cd exposure.

In male mice during the early phase of exposure (12 h–72 h), Cd was primarily distributed in the intestines; at 12 h, the proportion of Cd in the intestines reached as high as 86%, significantly higher than the proportion in feces during the same period; a small amount of Cd was also retained in the stomach at this time. As the exposure period prolonged, the proportion of Cd in the gut continued to decline, while the proportion in feces gradually increased, peaking at 14–21 days. Regarding distribution among target organs, Cd loading in the liver showed a marked upward trend between 28 and 35 days, while the proportion of Cd in the kidneys increased slowly over time; the proportion of Cd in other tissues remained at extremely low levels at all time points.

In female mice exposed during the early phase (12–72 h), Cd loading in the intestine was also overwhelmingly dominant. Over time, the proportion of Cd in the intestine gradually decreased, while the proportion in feces gradually increased; particularly in the later phase (28–35 days), feces accounted for the highest proportion of Cd loading outside the intestine.

Given that this study collected fecal samples only within 2 h prior to the mice’s euthanasia, it is difficult to precisely quantify the total fecal excretion during the 28-day exposure period using a simple summation method. Therefore, we used a mass balance model to further elucidate the overall excretion characteristics and distribution patterns of Cd in male and female mice. Based on the experimental data, during the 28-day exposure period, the total Cd intake per female mouse was 473.2 µg, whereas the total accumulation in various tissues (excluding feces and urine) at the end of the experiment was only 0.899 µg per mouse. The total Cd intake for male mice was approximately 893.8 µg, with a total accumulation of 0.818 µg per mouse. It can therefore be inferred that during the 28-day dietary exposure period, female and male mice excreted as much as 472.3 µg and 892.9 µg of Cd, respectively, via feces and urine, accounting for more than 90% of total intake. From the perspective of macroscopic mass balance, this result confirms that, despite individual differences in food intake, dietary Cd has extremely low bioavailability in the body; more than 90% of the Cd is rapidly excreted rather than retained long-term, with only trace amounts accumulating in tissues such as the liver and kidneys.

### 3.5. Total Cd Accumulation and Distribution in Mice

**Figure 5:**
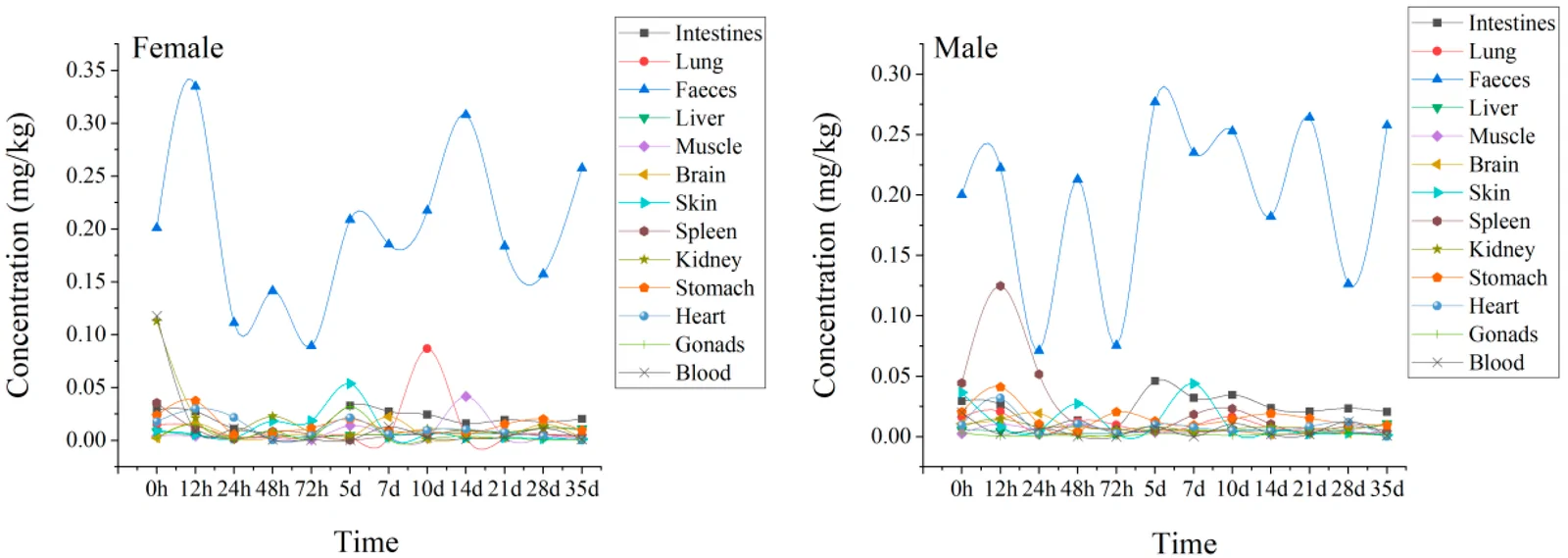
Concentrations in various tissues and faeces of control mice at different exposure times.

Figure 5 shows the Cd levels measured in various tissues and feces of the control group mice at different time points. At most time points, Cd concentrations in the tissues of the control group mice were below the limit of quantification (0.05 mg/kg), while Cd levels in feces were consistently the highest, indicating that Cd ingested through the standard diet is primarily excreted via feces. Throughout the 35-day experimental period, although trace amounts of Cd were occasionally detected in the kidneys, spleen, heart, blood, gonads, intestines, stomach, lungs, and fur of the control group mice, none of these showed a trend of persistent detection.

In the exposed group of mice, the diet first reached the stomach after oral ingestion. Although high levels of Cd were detected in the stomach during the exposure phase, the results from Day 35 indicated that the Cd in the stomach primarily originated from residual exposed diet that had not been completely emptied. After being digested in the stomach, food residues enter the intestine; therefore, higher concentrations of Cd can be detected in the intestine during the exposure period. After exposure ended and the animals were returned to a standard diet, Cd levels in the intestine decreased significantly but remained higher than those in the control group, which had been fed a standard diet throughout the study. These results indicate that, although some of the Cd detected in the intestine may be influenced by residual intestinal contents, the data from Day 35 suggest that the intestine itself can accumulate small amounts of Cd. Previous studies have shown that Cd in food can be absorbed into the body through the gastrointestinal tract. The mechanism primarily involves Cd competitively displacing metal ions such as Ca^2+^, Fe^2+^, and Zn^2+^ from their binding sites on membrane transporters, thereby altering cell membrane permeability and allowing Cd to enter the cell. Proteins involved in Cd absorption in the gastrointestinal tract primarily include DMT1, ZIP14, Ca^2+^-selective channels, and TRPV6; these proteins themselves serve as transmembrane transporters for essential elements such as Fe, Zn, and Ca [14]. Once Cd is absorbed and enters the bloodstream, it binds to thiol-rich plasma proteins and is rapidly distributed throughout the body via the bloodstream to various tissues and organs [15]. However, there is currently no definitive research conclusion as to whether the gastrointestinal tract retains some Cd or serves merely as a transit route for it. This study found that trace amounts of Cd were detected in the kidneys and livers of mice at 12 h and between 7 and 35 days, with concentrations showing a gradual increase over time, suggesting that Cd may accumulate in the kidneys and liver. These findings are consistent with previous reports [16]. Although trace amounts of Cd were detected in other tissues and organs of the mice—including the brain, lungs, spleen, fur, heart, and blood—at certain time points, they were not detected consistently, a pattern similar to that observed in the control group. It is hypothesized that Cd may temporarily reach these sites after entering the body but, due to mechanisms that have not yet been fully elucidated, is unable to accumulate effectively in these tissues. Further research is needed to determine how Cd enters the aforementioned tissues and organs and to identify the specific pathways of its migration and transport.

According to reports, after mice consume Cd-containing feed, Cd enters the body through the digestive tract and is rapidly distributed to various tissues and organs via the bloodstream [17]. However, in this study, no Cd was detected in the blood 12 h after exposure, whereas Cd levels in the kidneys and liver were actually higher than those observed at 48 h. Previous studies have suggested that blood Cd levels can accurately reflect Cd exposure status and are suitable as biomarkers for short-term exposure [18]. However, other studies have yielded results similar to those in this paper. Liu Ruying [19] found that when rats were fed Cd-contaminated brown rice, Cd was significantly absorbed in the gastrointestinal tract and accumulated markedly in the kidneys and liver, while only trace amounts of Cd were detected in whole blood samples. Combining the results of this study with those of the aforementioned literature, two hypotheses can be proposed: First, Cd absorbed through the gastrointestinal tract may bypass the bloodstream and enter the liver or kidneys directly via mechanisms related to the “enterohepatic circulation”; second, the transport of Cd in the blood may be extremely rapid, enabling the liver and kidneys to capture it immediately and efficiently. It is worth noting that previous studies on Cd exposure have primarily involved direct exposure of mice or rats to CdCl_2_, with exposure routes typically including drinking water [18], intraperitoneal injection, or gastric lavage [20]. In contrast, both this study and the aforementioned research involved dietary exposure, and the chemical forms of Cd were no longer limited to inorganic Cd, suggesting that Cd speciation may have a significant impact on the processes of absorption, accumulation, and excretion in mice. At the same time, this also suggests that relying solely on blood Cd levels to assess Cd accumulation may not be reliable; a comprehensive evaluation incorporating additional indicators and conditions is necessary.

In this experiment, most of the Cd exposure originated from the edible tissues of the swimming crab mixed into the feed; more specifically, it came primarily from Cd accumulated in the hepatopancreas of the swimming crab. A study by Zhao et al. [21] indicated that Cd in the hepatopancreas of the swimming crab mostly exists in the form of low-toxicity organic Cd; when total Cd content is high, it primarily exists as Cd-MT, while at lower concentrations, it coexists mainly as Cd-Cys and Cd-MT. To better reflect everyday dietary habits, in this experiment, mixed tissues from swimming crab were dried in an oven at 100 °C, ground, and then mixed with standard feed to produce pellet feed. During this processing, the chemical form of Cd may have changed. However, since there are no established detection methods for specific Cd forms, it is not possible to confirm the exact forms of Cd present in the feed.

## 4. Conclusions

In this study, a mouse model was established to investigate the accumulation, tissue distribution, and post-exposure elimination characteristics of Cd derived from Cd-enriched swimming crabs following a 28-day dietary exposure and a 7-day washout period. The results indicated that Cd retention from the swimming crab matrix was limited during the exposure period. More than 90% of the ingested Cd was not retained in the analyzed tissues, suggesting a low systemic retention efficiency; however, the kidneys and liver showed the highest Cd accumulation among the examined tissues. Even after a 7-day washout period with a standard diet, residual Cd was still detectable in these organs, indicating relatively slow clearance of retained Cd.

During the clearance period, kidney Cd concentrations in male mice showed a decreasing trend, whereas female mice exhibited a slight increasing trend. However, because male and female mice received different Cd exposure levels, these differences cannot be used to draw conclusions regarding sex-specific regulatory mechanisms. Further studies are needed to clarify the dynamic transport of Cd between tissues and the mechanisms underlying its retention and clearance. This study provides experimental evidence regarding the toxicokinetic behavior of seafood-derived Cd and offers preliminary information for evaluating Cd exposure associated with crustacean seafood consumption.

## 5. Limitations and Future Directions

Due to limitations in detection methodologies, this study was unable to perform qualitative and quantitative analyses of the specific chemical species of organic and inorganic Cd (Cd) in feed and mouse tissues. Furthermore, toxicological endpoints such as Cd-induced oxidative stress, inflammatory responses, and histopathological alterations were not evaluated, thereby precluding a comprehensive assessment of Cd-associated health risks. Accordingly, future research should prioritize the establishment of robust methodological frameworks for Cd speciation analysis to elucidate the toxicokinetic disparities between organic and inorganic Cd, particularly regarding their differential absorption in the gastrointestinal tract, transport in circulation, and accumulation in target tissues. Concurrently, integrative multi-omics approaches, including transcriptomics and proteomics, should be employed to systematically decipher the molecular mechanisms underlying Cd accumulation in the kidney and liver, as well as the perturbation of associated toxicological pathways. Additionally, investigating the antagonistic effects and optimal ratios of dietary nutrients including calcium (Ca), copper (Cu), magnesium (Mg), zinc (Zn), and iron (Fe) on Cd intestinal absorption and tissue accumulation would facilitate the development of precision dietary intervention strategies. Where feasible, population-based epidemiological investigations are warranted to validate the extrapolation of findings from animal models to human populations, thereby providing more robust scientific evidence for risk assessment and regulatory standard setting regarding Cd exposure from seafood consumption.

## Figures and Tables

**Table 1:** Procedure of microwave digestion.

Procedure	Rising Time/min	Holding Time/min	Temperature/°C	Power/W
Heating	15	/	180	1200
Digestion	/	30	180	1200

## Data Availability

The original contributions presented in this study are included in the article material. Further inquiries can be directed to the corresponding authors.
